# Viability and In Vitro Gastrointestinal Transit Tolerance of Multispecies Probiotic Combinations Incorporated into Orange Juice and Drinking Water

**DOI:** 10.3390/foods12112249

**Published:** 2023-06-02

**Authors:** Mahta Moussavi, Javad Barouei, Craig Evans, Michelle C. Adams, Surinder Baines

**Affiliations:** 1 Cooperative Agricultural Research Center, Prairie View A&M University, Prairie View, TX 77446, USA; 2 Integrated Food Security Research Center, Prairie View A&M University, Prairie View, TX 77446, USA; 3 School of Environmental and Life Science, The University of Newcastle, Callaghan, NSW 2308, Australia; 4 School of Health Sciences, The University of Newcastle, Callaghan, NSW 2308, Australia

**Keywords:** probiotic combinations, orange juice, viability, gastrointestinal transit tolerance

## Abstract

Little is known about how combining probiotics affects the storage survival and functional performance of individual probiotics when incorporated into non-dairy drinks. Viability of *Lacticaseibacillus rhamnosus* GG (LG), *Limosilactobacillus reuteri* ATCC 55730 (LR), *Bifidobacterium animalis* subsp. *lactis* BB-12 (Bb), and *Propionibacterium jensenii* 702 (PJ), either alone or in multi-species combinations included in orange juice (OJ), were assessed during storage in refrigerated conditions and compared with bottled water (BW). The tolerance of probiotics included in refrigerated OJ to simulated gastrointestinal conditions was also examined. LG and LR viabilities were significantly higher in OJ than in BW (*p* ≤ 0.001), while the reverse was evident for PJ. Bb maintained high viability in both drinks. LG-PJ in both drinks and Bb-PJ in BW resulted in greater viabilities among the paired combinations compared to their respective monocultures when incorporated separately (*p* ≤ 0.001). The viability of LG in the LG-Bb-PJ combination improved significantly in BW compared with LG alone (*p* ≤ 0.001). OJ did not alter bacterial tolerance to simulated gastric juice but diminished tolerance to simulated intestinal juice (SIJ). In all combinations, tolerance of LG and LR to SIJ was improved, whereas tolerance of PJ declined significantly compared with respective monocultures (*p* ≤ 0.001). In conclusion, probiotic storage stability and gastrointestinal transit tolerance were species-dependent and affected by carrier type and combinations. These effects should be considered when formulating probiotic products.

## 1. Introduction

Probiotics have been exploited primarily in the form of commercial dairy-based products such as fermented milk products and yogurt [1]. Development of dairy-free probiotic foods may, however, suit consumers who are allergic to specific milk components, are lactose intolerant [2], or have no desire to eat dairy foods due to dietary or sensory reasons. As an alternative to dairy foods, fruit juices may be considered ideal delivery vehicles for probiotics. However, intrinsic factors such as low pH values, low protein content, and the presence of natural antimicrobials in fruit juices may adversely affect the viability of probiotics. A minimum level of 10^6^–10^7^ CFU of viable probiotics per gram or milliliter of a probiotic product [3] or 10^9^ CFU per day, as regulated in some countries, is recommended at the consumption point [4]. It is, therefore, important that the viability of the probiotics remains optimal throughout the anticipated shelf life of probiotic products [5].

The viability of single strains of probiotics has been studied in fruit juices and fruit drinks, in which their survivability was shown to be genus, species, or strain dependent; viability is also affected by the type of fruit juice and its intrinsic parameters such as pH and the presence of particular compounds, and extrinsic factors such as storage temperature and duration [6,7,8,9,10].

For beneficial probiotic effects that are dependent on the viability and activity of probiotics in the intestine, it is critical that they survive hostile conditions of the upper gastrointestinal tract, such as gastric acid and exposure to bile salts and proteolytic enzymes in the duodenum [11]. Prior studies have shown that simulated gastrointestinal transit tolerance of probiotic bacteria is strain-, pH- [1], and food matrix-dependent [12,13]. It has also been speculated that due to the short gastrointestinal transit time of fruit juices, inclusion in such carriers may reduce exposure of probiotics to the harsh gastrointestinal environment [14]. The inclusion of probiotics in acidic fruit drinks could improve the tolerance of probiotic monocultures to simulated gastric juice (SGJ) through the preadaptation mechanisms [15]. The storage duration of probiotic fruit drinks is also another factor that may affect the tolerance of incorporated probiotic cultures to simulated gastrointestinal conditions [7,8].

Since probiotic organisms vary in the type and level of their health-promoting effects, it is likely that probiotic combinations may potentially offer even greater benefits to the consumer than single-strain preparations [16]. As such, consideration must be given to the potential occurrence and impact of interactions between individual probiotics within a proposed combination. Findings in relation to the inclusion of mixed cultures in carriers such as fermented dairy products have shown viability interactions between individual probiotics to be evident during storage [16]. It is not yet known whether long-term exposure to non-dairy delivery carriers impacts on the viability and gastrointestinal transit tolerance of individual strains in probiotic combinations.

The objective of the present study is to examine the viability and in vitro gastrointestinal transit tolerance of multi-species probiotics incorporated into orange juice and drinking water during storage under refrigeration (both carriers) or at room temperature (only drinking water). Orange juice was used as it is the most popular fruit beverage worldwide [17] and has been shown to support some probiotic monocultures at reasonable, viable cell levels during storage and consumer acceptance [7,9,18,19,20,21].

## 2. Materials and Methods

**Figure 1:**
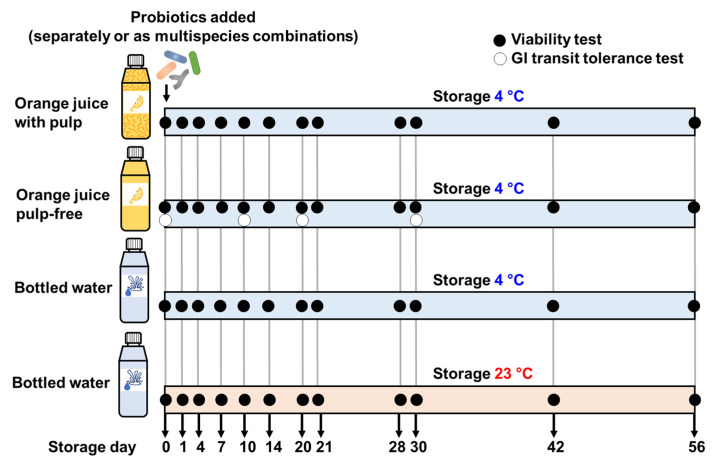
A timeline schematic of experimental procedures. Orange juice with and without pulp and bottled drinking water were supplemented with probiotics either with single-strain probiotics or multi-species probiotic combinations and stored at 4 °C (orange juices and drinking water) or 23 °C (only drinking water). Bacterial viability and tolerance to simulated gastrointestinal conditions were examined.

A timeline schematic of experimental procedures is presented in Figure 1.

### 2.1. Bacterial Cultures and Growth Conditions

*Lacticaseibacillus rhamnosus* GG (here referred to as LG, isolated from CULTURELLE^®^ capsule, Amerifit Brands Inc., Cromwell, CT, USA), *Limosilactobacillus reuteri* ATCC 55730 (here referred to as LR, BioGaia Biologics Inc. Raleigh, NC, USA) and *Bifidobacterium animalis* subsp *lactis* BB-12^®^ (here referred to as Bb, Chr. Hansen Pty. Ltd. Melbourne, Australia), as well as probiotic candidate *Propionibacterium jensenii* 702 (here referred to as PJ, isolated from raw cow’s milk in the Laboratory of Microbiology, School of Environmental and Life Science, The University of Newcastle, Newcastle, Australia) were used in this study. *Lactobacillus* strains (LG and LR) and Bb were grown overnight at 37 °C in MRS and RCM broths (Oxoid Australia Pty Ltd., Adelaide, Australia), respectively. PJ was grown in yeast extract lactate (YEL) [22] at 30 °C for 48 h.

### 2.2. Probiotic Drinks Preparation

A commercial blend of Navel and Valencia orange juice (OJ) with pulp (The Original Juice Co., a division of Golden Circle Ltd., Mill Park, VIC, Australia), pulp-free OJ prepared from the commercial OJ with pulp (pulp removed by centrifugation at 3220× *g* for 15 min at 4 °C), and bottled spring drinking water (here referred to as BW, Mount Franklin, Coca-Cola Amatil Pty, Ltd., Sydney, Australia) were used in this study. The OJ with pulp is composed of 10.8% carbohydrates, including 8.6% sugars, 0.7% protein, 0.1% total fat, sodium 1 mg 100 mL^−1^, and vitamin C 20 mg 100 mL^−1^. Fifty (50) mL aliquots of the OJ with and without pulp) and BW were then dispensed into sterile screw cap high-density polypropylene containers (Sarstedt AG & Co. KG, Nümbrecht, Germany) and refrigerated (4 °C) prior to further preparation. Bacterial cells were grown and harvested in their stationary phases by centrifugation (3220× *g*, 15 min, 4 °C) and washed three times with PBS, pH 7.0 (Gibco, Invitrogen Corp., Carlsbad, CA, USA). Viable bacterial cells were enumerated in PBS culture stocks and after adding to the products to ensure the viable cell counts for each species remained constant across the preparations. An aliquot of each bacterial suspension in PBS was incorporated into the 50 mL portions of the carriers to achieve final concentrations of ~1 × 10^8^, ~1 × 10^8^, ~1 × 10^7^, and ~3 × 10^8^ live cells mL^−1^ of the product for LG, LR, Bb, and PJ, respectively. Eleven preparations were made for each carrier as follows, LG, LR, Bb, PJ, LG + Bb, LG + PJ, LR + Bb, LR + PJ, Bb + PJ, LG + Bb + PJ, and LR + Bb + PJ (see Table 1 for bacterial formulations). The containers were then stored at 4 °C for a period of 8 weeks (Figure 1). A complete set of BW preparations prepared with the 11 bacterial formulations was also stored at 23 °C (Figure 1).

### 2.3. Viability of Probiotics in the Drinks

Bacterial viable cell counts were determined by plating of decimal dilutions prepared from OJ and BW at storage days 0, 1, 4, 7, 14, 21, 28, 42, and 56 (Figure 1). Lactobacilli, either alone or in combination with PJ, were enumerated on lactobacillus selection (LBS) agar [23] and incubated anaerobically at 37 °C for 72 h. To ensure inhibition of Bb growth, lactobacilli, in combination with Bb, were counted on MRS agar supplemented with 1 mg mL^−1^ Vancomycin (Sigma-Aldrich, St. Louis, MO, USA) (MRS-V) after anaerobic incubation of the plates at 43 °C for 24 h [24]). PJ was counted on YEL agar after anaerobic incubation at 30 °C for 7 days. Bb was counted on TOS propionate agar (Yakult Pharmaceutical Ind., Co., Ltd., Tokyo, Japan) following anaerobic incubation of the plates at 37 °C for 2 days. The results were expressed as Log CFU mL^−1^.

### 2.4. Measurement of pH, Titratable Acidity, and Brix of Drinks

pH values of the OJ and BW were measured on the same days as the survival determinations. Titratable acidity of orange juices was determined in the beginning (time point 0) and at the end of refrigerated storage (week 8) by titration of the juices with 0.1 N NaOH to the phenolphthalein endpoint (pH 8.2) also monitored using a pH-meter. Total soluble solids or Brix was determined by the refractometry method of the Association of Analytical Chemists [25] using a refractometer model REF113 (Bacto Laboratories Pty Ltd., Liverpool, Australia).

### 2.5. In Vitro Gastrointestinal Transit Tolerance Assay

Due to technical difficulties (frequent pipette tip blockage) with orange juice containing pulp, we performed a GI transit tolerance test only on pulp-free OJ products. The test was conducted by exposing probiotic pulp-free OJ preparations to simulated gastric juice (SGJ) and simulated intestinal juice (SIJ) at storage days 0, 10, 20, and 30 (Figure 1). SGJ was prepared by adding pepsin (~2500 units/mg, Chem-supply Pty Ltd., Gillman, SA, Australia) to a final concentration of 3.0 g L^−1^ to sterile PBS. The pH value was then adjusted to pH 2.0 with 0.2N HCl (Sigma-Aldrich). SGJ tolerance was determined by adding an aliquot of 1.0 mL probiotic OJ to 9 mL SGJ followed by vortex mixing for 10 s and incubation at 37 °C [26] for 20 min—the mean residence time of OJ in the stomach [27]. Immediately after incubation, the suspension was vortex mixed briefly. One mL of the suspension was then transferred to a tube containing 9 mL of sterile Maximum Recovery Diluent (Oxoid). Bacterial counts were determined as described above in Section 2.3.

To determine the tolerance of probiotics to the simulated intestinal conditions, the method of de Palencia et al. [28] was followed, with a slight modification by increasing the incubation time from 120 min to 180 min. Briefly, SIJ was prepared by dissolving porcine pancreatin (Sigma-Aldrich) in PBS to a final concentration of 1.0 g L^−1^. The mixture was then centrifuged at 2000× *g* for 15 min at 4 °C. Bile Salts No 3 (Sigma-Aldrich) was added to a final concentration of 3.0 g L^−1^ to the decanted supernatant and the pH adjusted to 8.0 with 1.0N NaOH (Sigma-Aldrich). Tolerance to the intestinal conditions was determined by transferring a 2.5 mL aliquot of each probiotic OJ preparation to 7.5 mL of a mixture of SGJ (45 mL), SIJ (60 mL), and 1.0M NaHCO_3_ (6.1 mL). The final mixture was vortex mixed for 10 s and incubated at 37 °C for 180 min. The viable bacterial cell counts were then determined, as described in Section 2.3.

### 2.6. Statistical Analysis

Statistical analyses were performed using SPSS software Ver. 18 (SPSS Inc., Chicago, IL, USA). Data were analyzed using the linear mixed model procedure to run a general linear model (GLM). In order to give protection against potential false positives (false significant results), the Bonferroni adjustment was used, and the significance level (α) was set at *p* ≤ 0.001.

## 3. Results

### 3.1. Viability of Probiotics in the Carrier Drinks during Storage

#### 3.1.1. Orange Pulp and Probiotic Viability

Only 4 out of 11 OJ preparations showed significant differences between the bacterial viabilities in OJ with and without pulp (*p* ≤ 0.001) (Supplementary Figure S1). Where a significant variation was observed, viability was found to decline more rapidly in the OJ containing pulp. Compared with pulp-free OJ, significant reductions were observed in the viabilities of LR monoculture and Bb in the LG-Bb combination included in OJ containing pulp after week 4 (*p* ≤ 0.001). A similar result was observed for Bb in the Bb-PJ combination and for PJ in the LG-PJ combination after week 2 and week 6, respectively (Supplementary Figure S1).

#### 3.1.2. Strain and Carrier Dependent Variation in Probiotic Viability

**Figure 2:**
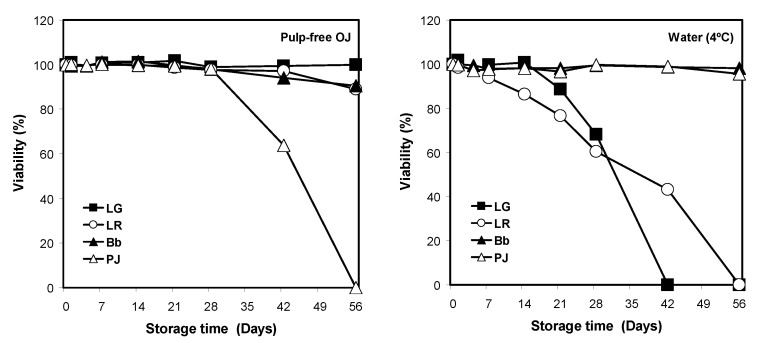
Viability percentage of probiotic monocultures in pulp-free orange juice (OJ) and bottled water (BW) over 8 weeks of storage at 4 °C. Due to the smaller size of standard error (SE) values compared with the size of markers, they are hidden behind the markers.

Apart from LR monoculture, insignificant differences were observed between the survival of probiotic monocultures included in OJ, either with or without pulp. Hence, the results on strain-dependent differences in viability are presented in pulp-free OJ (Figure 2). In order to standardize the comparisons of viability, the bacterial counts at each time point throughout the storage period have been expressed as a percentage of the initial count for each strain.

Among the four monocultures in pulp-free OJ, LG had the highest viability, which remained constant throughout the entire storage period (Figure 2). The viabilities of LR and Bb monocultures were also maintained in OJ with cell counts expressed as Log CFU mL^−1^, reducing by about 10% across the 8 weeks of storage (Figure 2). By comparison, the viability of PJ in OJ declined steeply after the first 4 weeks, with no viable cells recovered at the end of storage.

In refrigerated BW, Bb and PJ were the most stable of the probiotics examined, with cell counts of both remaining relatively unchanged throughout the storage (Figure 2). In contrast, the viability of LG and LR declined rapidly in BW, with no viable cells recovered by day 42 and 56, respectively. LG and LR counts were significantly lower in BW than in OJ at each time point beyond days 21 and from day 7, respectively (*p* ≤ 0.001) (Figure 2).

#### 3.1.3. Storage Temperature and Probiotic Viability in Bottled Drinking Water

**Figure 3:**
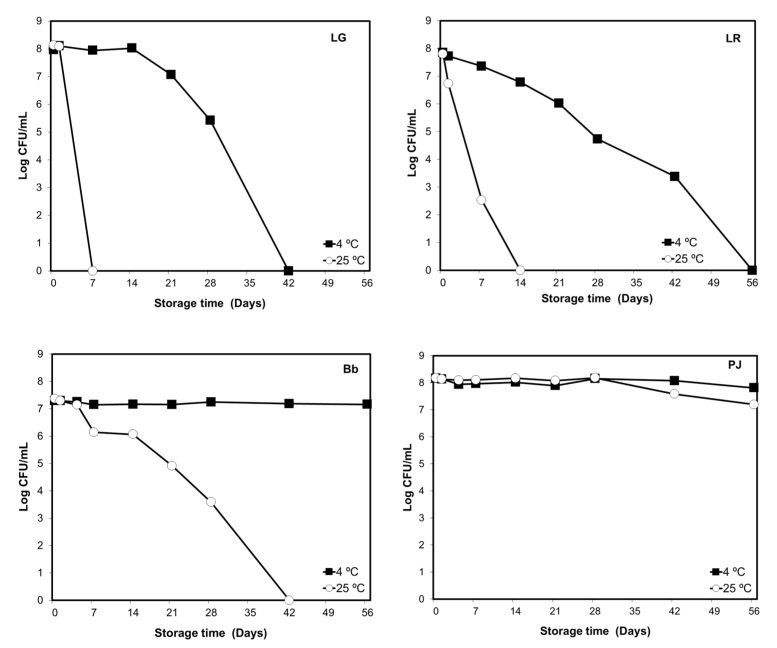
Viable cell counts of probiotics in bottled water supplemented with single probiotic cultures LG, LR, Bb, and PJ over 8 weeks of storage at 4 °C (■) and 23 °C (○). The results are presented as mean ± SE Log CFU mL^−1^. Due to the smaller size of standard error (SE) values (ranging from 0.004 to 0.120 Log CFU mL^−1^) compared with the size of markers, they are hidden behind the markers.

In refrigerated BW (4 °C), *Lactobacillus* viabilities were found to remain above the effective threshold level (10^6^ CFU mL^−1^) up to 4 weeks of storage, but at room temperature (23 °C), their viabilities dropped to zero within 2 weeks of storage (Figure 3). Bb viability remained relatively stable in the refrigerated BW but decreased sharply under non-refrigerated storage such that no viable cells were recovered after week 6 (Figure 3). In contrast, PJ maintained a high level of viability throughout the storage period in both refrigerated and non-refrigerated BW (Figure 3).

#### 3.1.4. Viability of Individual Strains in Probiotic Combinations

**Figure 4:**
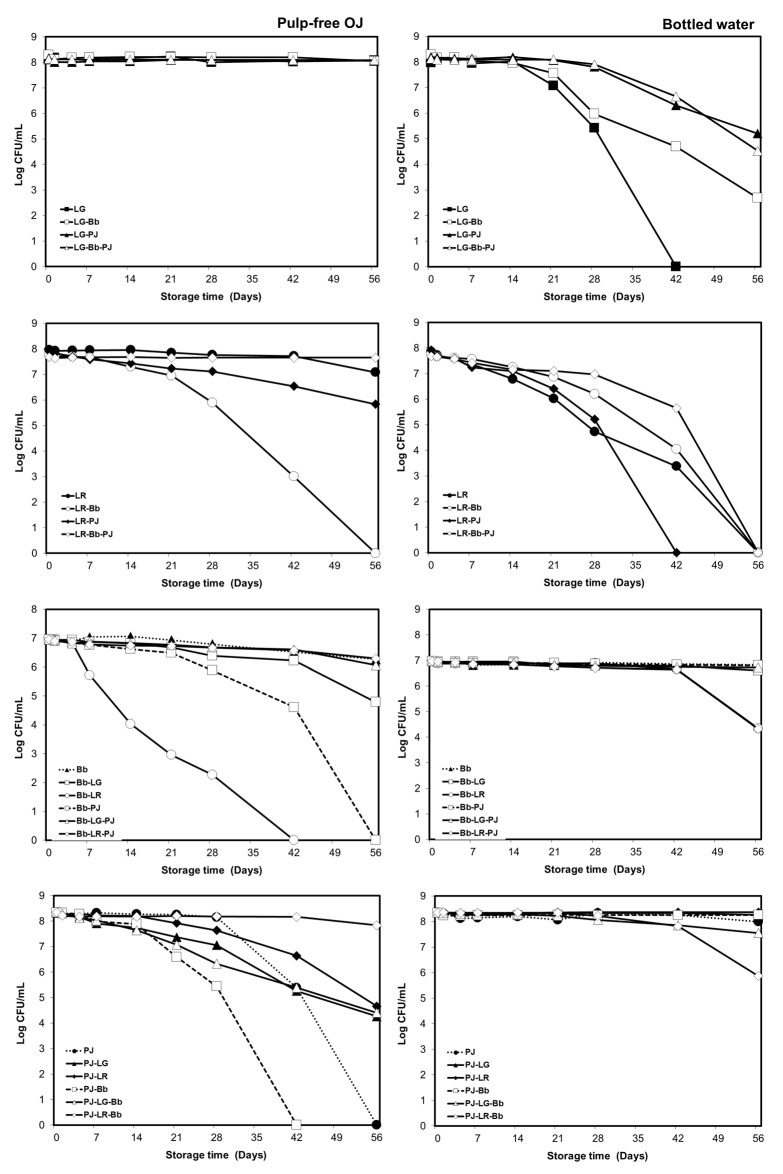
Viabilities of the four probiotics, both in monoculture and in the designated combinations, in pulp-free orange juice (OJ) and bottled water over 8 weeks of storage at 4 °C. In combinations, the cell counts refer to the first listed bacterium. The results are presented as mean ± SE Log CFU mL^−1^). Due to the smaller size of standard error (SE) values (ranging from 0.003 to 0.077 Log CFU mL^−1^) compared with the size of markers, they are hidden behind the markers.

Due to little if any difference between the results obtained in OJ with or without pulp (see Section 3.1.1 and Supplementary Figure S1) and poor viability in BW stored at 23 °C (except PJ), and for the purposes of clarity, the results obtained in pulp-free OJ, and BW stored at 4 °C are presented in this section (Figure 4).

In pulp-free OJ, the viability of the LG was not affected by the presence of Bb and/or PJ. When combined, LR and Bb viabilities declined significantly, as was the case for the Bb-PJ combination. In contrast, the triplet combination (LR-Bb-PJ) produced no decline in the viability of either LR or Bb and a significant enhancement to the viability of PJ, which in this case remained constant throughout the entire 8-week storage (Figure 4).

In OJ, the viability of PJ improved in all combinations except for Bb-PJ compared with PJ monoculture. However, LR viability declined significantly in the LR-PJ combination (*p* ≤ 0.001).

Similarly, in refrigerated BW, LG viability was enhanced in all combinations (Figure 4). The viability of LR was improved in LR-Bb, and LR-Bb-PJ combinations but diminished significantly after day 28 in the LR-PJ combination compared with LR monoculture (*p* ≤ 0.001). In the BW, the LR-Bb and LR-Bb-PJ combinations appeared to be slightly less favorable for both Bb and PJ, although in these cases, decreases in viability were only evident beyond day 42. In all other cases, in both OJ and BW, the bacterial viability appeared to be largely unaffected by the presence of other probiotic species (Figure 4).

### 3.2. pH and Brix Changes in Orange Juices

The initial pH values of OJ with and without pulp were 3.81 ± 0.01 and 3.82 ± 0.01, respectively. The value for drinking water was 6.60. The pH (ranged from 3.73–3.83), titratable acidity (ranged from 1.15–1.22%), and Brix (ranged from 10.3–10.7) values of OJ preparations, and pH values of water (under refrigeration) were relatively constant across the entire data set.

### 3.3. Effect of Pulp-Free Orange Juice on Simulated GI Tolerance of Probiotics

**Figure 5:**
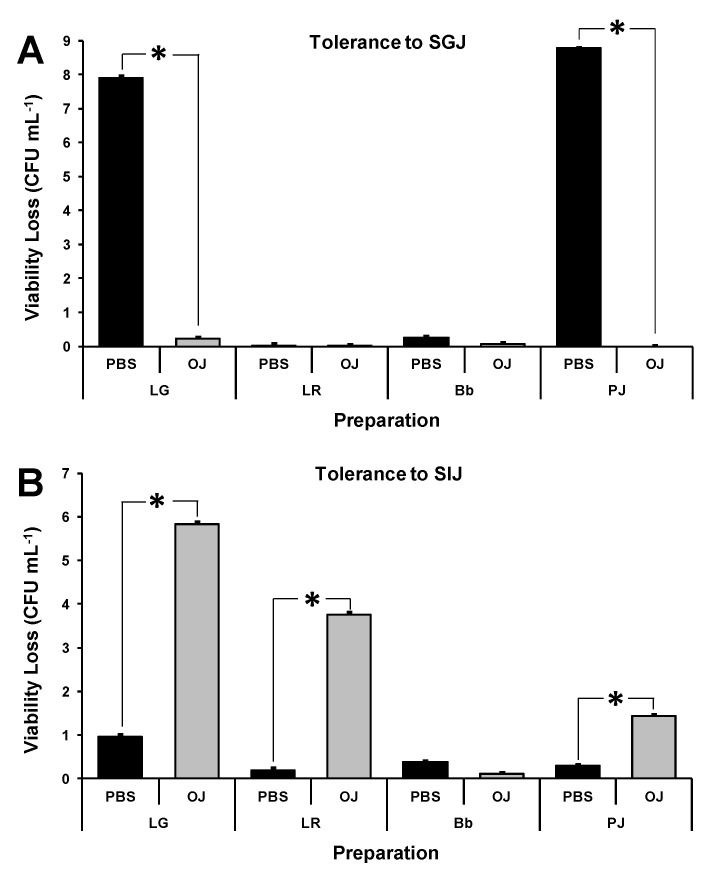
Reduction in the viability of individual probiotic *Lacticaseibacillus rhamnosus* GG (LG), *Limosilactobacillus reuteri* ATCC 55730 (LR), *Bifidobacterium animalis* subsp *lactis* BB-12 (Bb) and *Propionibacterium jensenii* 702 (PJ) strains incorporated into phosphate-buffered saline (PBS) or orange juice (OJ) at day 0 after exposure to (**A**) simulated gastric juice (SGJ) for 20 min at 37 °C or (**B**) simulated intestinal juice (SIJ) for 180 min at 37 °C. Viability losses were determined as the difference between the viable bacterial count of the preparation pre- and post-exposure to SGJ or SIJ (expressed as Log CFU mL^−1^). An asterisk (*) shows a significant difference (*p* ≤ 0.001).

The inclusion of probiotics in OJ did not adversely affect the survival of the bacterial strains when exposed to SGJ, with the tolerance of LG and PJ significantly enhanced relative to the control PBS (*p* ≤ 0.001) (Figure 5A). However, inclusion in OJ was associated with significant decreases in the tolerance of the lactobacilli to SIJ compared with PBS (*p* ≤ 0.001) (Figure 5B). When incorporated into OJ, PJ exhibited a strong tolerance to SGJ and a relatively moderate intolerance to SIJ. Bb maintained its full viability in all scenarios (Figure 5).

### 3.4. Effect of Probiotic Combinations and Storage in Pulp-Free OJ on Simulated GI Tolerance

**Figure 6:**
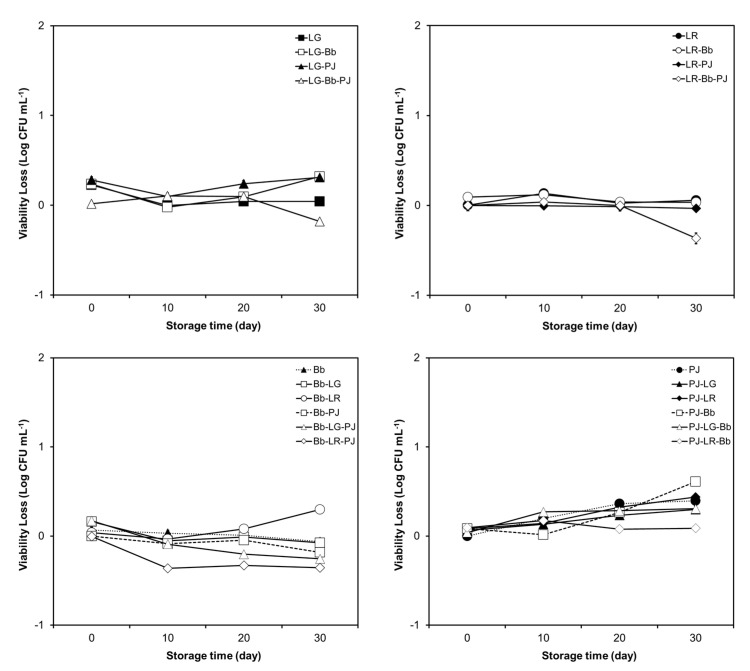
Variation in the tolerance of *Lacticaseibacillus rhamnosus* GG (LG), *Limosilactobacillus reuteri* ATCC 55730 (LR), *Bifidobacterium animalis* subsp *lactis* BB-12 (Bb), and *Propionibacterium jensenii* 702 (PJ) to simulated gastric conditions, in monoculture, and in multi-species combinations, during 30 days of refrigerated storage (at 4 °C) in orange juice (OJ). The results are presented as a reduction in the number of viable cells after exposure to simulated gastric juice (SGJ) (expressed as mean ± SE Log CFU mL^−1^). Due to the smaller size of standard error (SE) values (ranging from 0.020 to 0.059 Log CFU mL^−1^) compared with the size of markers, they are hidden behind the markers.

**Figure 7:**
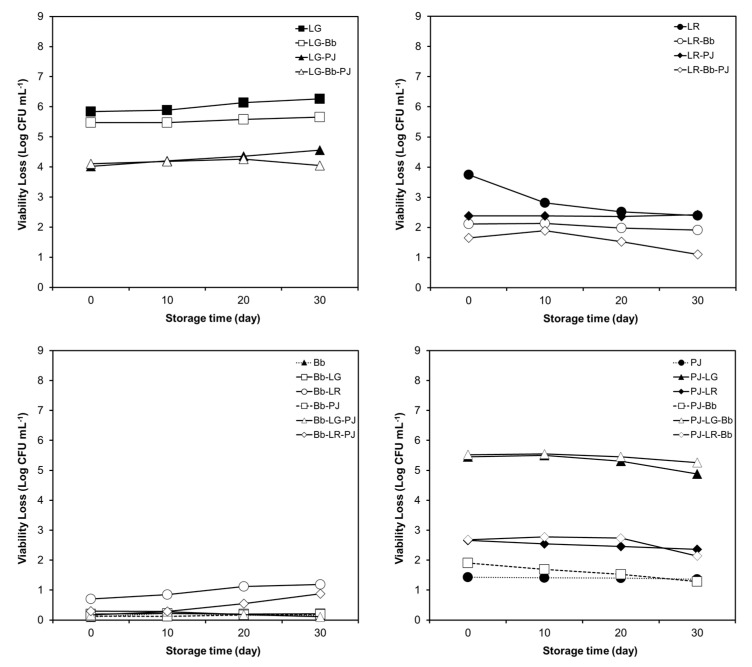
Variation in the tolerance of *Lacticaseibacillus rhamnosus* GG (LG), *Limosilactobacillus reuteri* ATCC 55730 (LR), *Bifidobacterium animalis* subsp *lactis* BB-12 (Bb), and *Propionibacterium jensenii* 702 (PJ)to simulated intestinal conditions, in monoculture, and in multi-species combinations, during 30 days of refrigerated storage (at 4 °C) in orange juice (OJ). The results are presented as the reduction in the number of viable probiotic cells after exposure to simulated intestinal juice (SIJ) (expressed as mean ± SE Log CFU mL^−1^). Due to the smaller size of standard error (SE) values (ranging from 0.020 to 0.059 Log CFU mL^−1^) compared with the size of markers, they are hidden behind the markers.

The results have been presented as two sets of figures (Figure 6 and Figure 7), one for the tolerance of probiotics to SGJ (Figure 6) and the other one for the tolerance of probiotics to SIJ (Figure 7). Each set contains separate plots of the tolerance of the four probiotics, either alone or in combinations. In order to ensure that any apparent variations in the GI transit tolerance were not confounded by general losses in viability due to cold storage, viable cell counts prior to exposure to SGJ and SIJ were determined at each time point.

A high tolerance of monoculture preparations to SGJ was maintained throughout the storage period (Figure 6). The presence of other probiotics (in combinations) had little or no impact on the tolerance of any of the individual probiotics to SGJ (Figure 6).

A steady reduction in the tolerance of LG was observed between the initial (day 0) and final (day 30) measurements (Figure 6).

In several cases, combining probiotics had a substantial impact on the intestinal transit tolerance of individual probiotics (Figure 7). For example, paired and triplet combinations involving LG and PJ appeared to simultaneously result in an approximate 100-fold improvement in the tolerance of LG to SIJ (Figure 7) but a 10,000-fold decline in the tolerance of PJ (Figure 7) compared with their LG and PJ monocultures, respectively. When combined with LR, the tolerance of PJ to SIJ also appeared to be adversely affected, although to a lesser extent relative to when combined with LG (Figure 7).

The viability loss of LR exposed to SIJ reduced significantly after 10 days of storage compared with day 0 and remained unchanged by the end of storage (Figure 7). The tolerance of LR to SIJ was significantly improved when combined with both PJ and Bb (Figure 7), although combining with LR resulted in a moderate reduction in the tolerance of Bb in paired and triplet combinations to SIJ as storage time increased (Figure 7). The tolerance of Bb to SIJ was unaffected in other combinations (Figure 7). Variations in intestinal transit tolerance across the storage period were also evident for individual strains in several of the other combinations; however, the magnitude of these variations was, in most cases, relatively insignificant within the context of initial viability losses (*p* ≤ 0.001).

In general, combining both Bb and PJ appeared to provide a favorable outcome for the lactobacilli with little impact on Bb, while the tolerance of PJ to SIJ was adversely affected to a varying extent in combinations with lactobacilli (Figure 7).

## 4. Discussion

Fruit juices have been considered a promising delivery vehicle for probiotics. However, maintaining the viability of probiotics incorporated into fruit juices during storage has been identified as the main limiting factor due to the low pH values of the products [29]. The present study reported that bacterial species incorporated as activated cultures [18], carrier matrix (orange juice or bottled drinking water), combining probiotics, storage duration, and storage temperature (for drinking water) all appeared to impact the survival of the probiotics. Furthermore, in most cases, pulp did not affect the survival of probiotics included in orange juice.

Refrigerated storage of fruit juices is required to prevent the growth and activity of microorganisms [30]. Consistent with previous studies, our results showed that the pH and titratable acidity values of the probiotic fruit juice were relatively constant during the refrigerated storage period [8,10,15]. This was the case for Brix values as well. While these results could potentially suggest very weak or no metabolic activity, such as assimilation/fermentation of sugars and production of organic acids by the bacteria, further analyses, such as profiling sugars and acids in the product, would be warranted.

Our results on species-dependent variation in bacterial viability are also in agreement with previous studies evaluating fruit juices as probiotic carriers [8,9], in which the low pH value of the juices was identified as the main determinant of survival [9,15]. Consistent with a higher tolerability of lactobacilli to lower pH values than bifidobacterial [31], and in agreement with prior studies, LG and LR were highly robust, and Bb was relatively stable for their capacity to remain viable in high numbers in the fruit juice throughout the storage period [8,9]. Bifidobacteria are normally sensitive to pH values < 4.6 [32]. However, *Bifidobacterium animalis* subsp *lactis* strains, including Bb, have been reported to be more resistant to low pH values [11]. In contrast, PJ exhibited considerable instability in orange juice. This is consistent with our prior study showing that PJ is more sensitive to low pH than lactobacilli, including LG and LR [33].

Apart from the pH, the higher survival of lactobacilli in orange juice than in drinking water is consistent with viability-promoting effects of organic components such as sugars [34], and antioxidants vitamin C [10,35], and hesperidin (the main flavonoid in orange juice) present in orange juice [36]. Previous studies have shown that the addition of antioxidants can result in improved survival of probiotics in fruit juices [29]. The natural antioxidants present in orange juice scavenge the oxygen present in the juice, thereby providing a more favorable anaerobic environment for the obligate or facultatively anaerobes such as bifidobacteria and lactobacilli.

Refrigerated drinking water did not support the survival of the *Lactobacillus* strains but did so for Bb and PJ. These results would certainly be associated with the differences in the compositional and physicochemical properties between orange juice and drinking water. The lack of organic compounds such as sugars and antioxidants in water could result in compromised viability for lactobacilli. The higher survival rate of PJ and Bb in water is likely due to entering a dormancy state induced by carbon starvation [37,38].

The effect of combining probiotics on the viability of individual strains appeared to be dependent on species, carrier matrix, and the composition of the probiotic combination. In paired combinations included in orange juice, the viability of at least one of the probiotics significantly declined, except for LG-PJ, in which PJ viability improved with no effect on LG. Interestingly, the viabilities of individual probiotics in the triplet combinations (LG-Bb-PJ, LR-Bb-PJ) were not adversely affected. In particular, PJ viability, which was poor in orange juice, was significantly improved in triplet combinations. It, therefore, was apparent that the adverse viability effects of pairing probiotics could be overcome by formulating triplet combinations. In drinking water, paired and triplet combinations comprising LG, Bb, and PJ appeared to have no adverse effects on the viabilities of Bb or PJ and significantly improved the viability of LG. Suboptimal growth temperatures, such as refrigerated storage, result in decreases in the metabolic activity of probiotics, thus extending the survival of probiotics [21,29]. Under refrigeration, the altered viability of individual probiotics in the combinations is more likely due to biophysical and/or biochemical interactions between microorganisms. We noted probiotics settled as a white to a creamy thin layer of precipitates at the bottom of the containers, which was not observed in the control drinks with no probiotics added. However, a quantitative method, e.g., turbidity measurement, would confirm the observation. This observation could result in the bacteria being in direct or closer physical contact with each other providing a protective bioshield by the constituents of the probiotic combinations against unfavorable conditions of the environment, such as low pH values. Additionally, this could result in bacterial coaggregation with other probiotic strains through biochemical interactions between the bacterial surface molecules [39,40,41]. Bacterial surface compounds and/or substances released by microorganisms may determine the potential mode of action of bacterial interactions on the viability of probiotic strains. However, mechanisms underlying the improved or compromised viability of probiotics in the presence of other probiotics warrant further investigation.

The present study also reports significant variation in the viability of probiotics in drinking water under different storage temperatures. When compared with refrigerated water, the decline in the viability of LG, LR, and Bb was found to be more dramatic under storage at room temperature. In spite of the suitability of room temperature for the growth of bacteria, the absence of carbon energy sources (i.e., metabolizable sugars), nitrogen sources, and other substances necessary for bacterial growth and maintenance in drinking water could result in starvation and, ultimately, bacterial death. Interestingly, PJ and Bb were much more stable than the lactobacilli in drinking water at both temperatures. Prolonged survival of propionibacteria has been reported when stored at room temperature under carbon starvation which may induce bacterial dormancy [37], thus extending the survival of propionibacteria. Dormancy has also been indicated as a survival mechanism utilized by bifidobacteria under stress conditions [38].

The presence of pulp in orange juice was not identified as an influential factor in the viability of probiotics in most of the probiotic preparations. However, where significant variations were observed, viability was found to decline more rapidly in the orange juice containing pulp. It has been shown that orange pulp contains essential oils, such as citrullene and limonene, which can exert a potent antimicrobial activity [42,43].

Based on the viability data, we also evaluated the shelf lives of the probiotic products. It has been recommended that the number of viable probiotic cells present in the food must be maintained above a minimum level of 10^6^ CFU mL^−1^ throughout the shelf life of the product [3]. According to the manufacturer of the orange juice used in this study, the product had a 38-day shelf life at 4 °C; therefore, probiotic orange juice preparations in which the minimum effective level of viable microorganisms was maintained for more than 42 days could be considered acceptable (Table 1). In the case of combinations, the shelf-life was determined as the number of days for which viable cell counts of all probiotic constituents remained above 10^6^ CFU mL^−1^. In most cases, pulp did not affect the shelf life of the probiotic orange juices. Orange juices containing monocultures of LG, LR, or Bb, paired combinations of LG-Bb and LR-PJ, and triplet combinations, all maintained the minimum effective level of viable cell counts of all probiotic constituents beyond week 6. In some cases (PJ monoculture and LG-PJ), the viability of at least one of the probiotic constituents dropped below the minimum recommended level between days 28 and 42. Since the exact day of declining below the effective threshold was unknown in these cases, they may be considered marginal.

In refrigerated drinking water preparations, both Bb and PJ and paired and triplet combinations containing both exhibited shelf-lives greater than 6 weeks. At room temperature, however, only PJ monoculture exhibited an acceptable shelf-life (Table 1).

In the present study, we also assessed in vitro gastrointestinal transit tolerance of probiotic combinations incorporated into orange juice. While not a requirement in pre-clinical assessments [44], survival in the harsh conditions encountered during gastrointestinal passage (digestive enzymes, bile, and acid) and then persistence in the intestinal environment [5] are critical for probiotic effects that are dependent on their viability and activity in the intestine [11]. Consistent with prior studies, our data showed that the tolerance of probiotics to simulated gastrointestinal conditions varies depending on the bacterial species/genus [11,26,45].

We also showed that the specific carrier matrix could impact the tolerance of the probiotics to simulated gastrointestinal conditions. The enhanced tolerance of LG and PJ included in orange juice to SGJ, compared to those included in PBS, is likely associated with the compositional and physicochemical properties of orange juice. It has been reported that components such as sugars and vitamin C that are present in orange juice can enhance the survival of probiotic lactobacilli and bifidobacterial in acidic environments [34,35]. Another possible mechanism for improved tolerance of LG and PJ to simulated gastric conditions when included in orange juice is bacterial adaptation to acid stress, whereby exposure to orange juice (pH 3.8) could improve bacterial resistance to subsequent more acidic conditions (SGJ, pH 2.0) The results are in agreement with prior studies reporting improved viabilities of preadapted probiotic lactobacilli and bifidobacteria [46,47,48] and propionibacteria [49] to mild or sublethal acidic conditions in subsequent more acidic conditions.

On the contrary, exposure to orange juice appeared to significantly decrease or have no effect on the tolerance of LG, LR, and PJ to SIJ. This result is consistent with previous studies where pre-exposure of lactobacilli, bifidobacterial, and propionibacteria to low pH values resulted in a decrease or no effect in their tolerance to simulated intestinal juice (1.4% bile in PBS) [48,49]. Lower tolerance of acid-adapted cells to SIJ is more likely due to enhanced bacterial susceptibility to bile salts which disrupt the bacterial membrane, denature proteins, and damage DNA.

The present study also showed that the duration of refrigerated storage of the probiotic species (either alone or in combinations) included in orange juice did not have an adverse impact on the bacterial tolerance to SGJ and SIJ over one month of storage compared with their tolerance at the baseline. Similar unchanged bacterial tolerance to SIJ was previously reported for probiotic lactobacilli included in a fruit juice blend [10]. However, this study showed that the tolerance of the bacteria to acid (pH 2.0 for 2 h) was significantly impaired. This apparent contrary finding may well be due to the shorter exposure time to SGJ in our study (20 min).

Lastly, we also showed that combining probiotics may favorably or adversely affect the tolerance of individual probiotics to SIJ but not SGJ. To the authors’ knowledge, this is the first report showing that the tolerance to intestinal conditions of probiotic strains included in a non-dairy carrier vehicle may be influenced by the presence of other strains. While this effect can be attributed to biophysical contact and/or biochemical interaction between the probiotics that may protect or have a detrimental effect on the tolerance of the bacteria to SIJ, potential underlying mechanisms are yet to be investigated.

## 5. Conclusions

The present study reports that the incorporation of probiotic combinations in non-dairy drinks may result in distinct outcomes in the performance of probiotics, such as viability and GI transit tolerance which could not be predicted based on findings from the inclusion of individual probiotics separately. However, additional studies are needed to further investigate the underlying mechanism of interactions. In light of a need for developing non-dairy probiotic products for consumers who are allergic or intolerant to milk components or have no desire to eat dairy foods, developing new probiotic food products with probiotic combinations should be carefully considered by the functional food industry so that the performance of individual probiotics including storage stability and tolerance to the gastrointestinal conditions is not, at least, compromised by the presence of other probiotics. Such products may also result in offering greater health-promoting effects to the consumer than single-strain preparations. However, the effect of the inclusion of probiotic combinations into non-dairy carrier vehicles on the sensory characteristics of the product and functional properties of probiotics in the digestive tract (such as adhesion to the intestinal epithelial cells, probiotic-microbiome and host cell-microbe interactions, neuroendocrine, and immune effects, etc.) needs to be further investigated.

## Figures and Tables

**Table 1:** Shelf-life (in days) of the probiotic drinks in terms of cell counts above the minimum effective level of viable microorganisms (≥10^6^ CFU mL^−1^) in orange juice (OJ) and bottled drinking water (BW). In combination, all components must meet the minimum requirement.

Probiotic(s)	Shelf-Life (Days)			
	Pulp Free OJ	OJ with Pulp	BW 4 °C	BW 23 °C
LG	>56	>56	21–28	4–7
LR	>56	42–56	14–21	4–7
Bb	>56	>56	>56	14–21
PJ	28–42	28–42	>56	>56
LG-Bb	42–56	28–42	28–42	0–4
LG-PJ	28–42	28–42	42–56	1–4
LR-Bb	7–14	7–14	28–42	4–7
LR-PJ	42–56	42–56	21–28	4–7
Bb-PJ	21–28	21–28	>56	7–14
LG-Bb-PJ	42–56	42–56	42–56	4–7
LR-Bb-PJ	>56	42–56	42–56	4–7

## Data Availability

Data is contained within the article or Supplementary Material.

## Supplementary Materials

The following supporting information can be downloaded at: https://www.mdpi.com/article/10.3390/foods12112249/s1, Figure S1: The viability of probiotics either separately or in combinations in orange juice with or without pulp.
