# Supplementary material for: Viability and In Vitro Gastrointestinal Transit Tolerance of Multispecies Probiotic Combinations Incorporated into Orange Juice and Drinking Water
Source: Foods. 2023 Jun 2;12(11):2249. doi: 10.3390/foods12112249 (PMC10252187; doi:10.3390/foods12112249)
Supplement: Supplementary file 1 [file foods-12-02249-s001.zip › foods-2418615-supplementary.pdf]

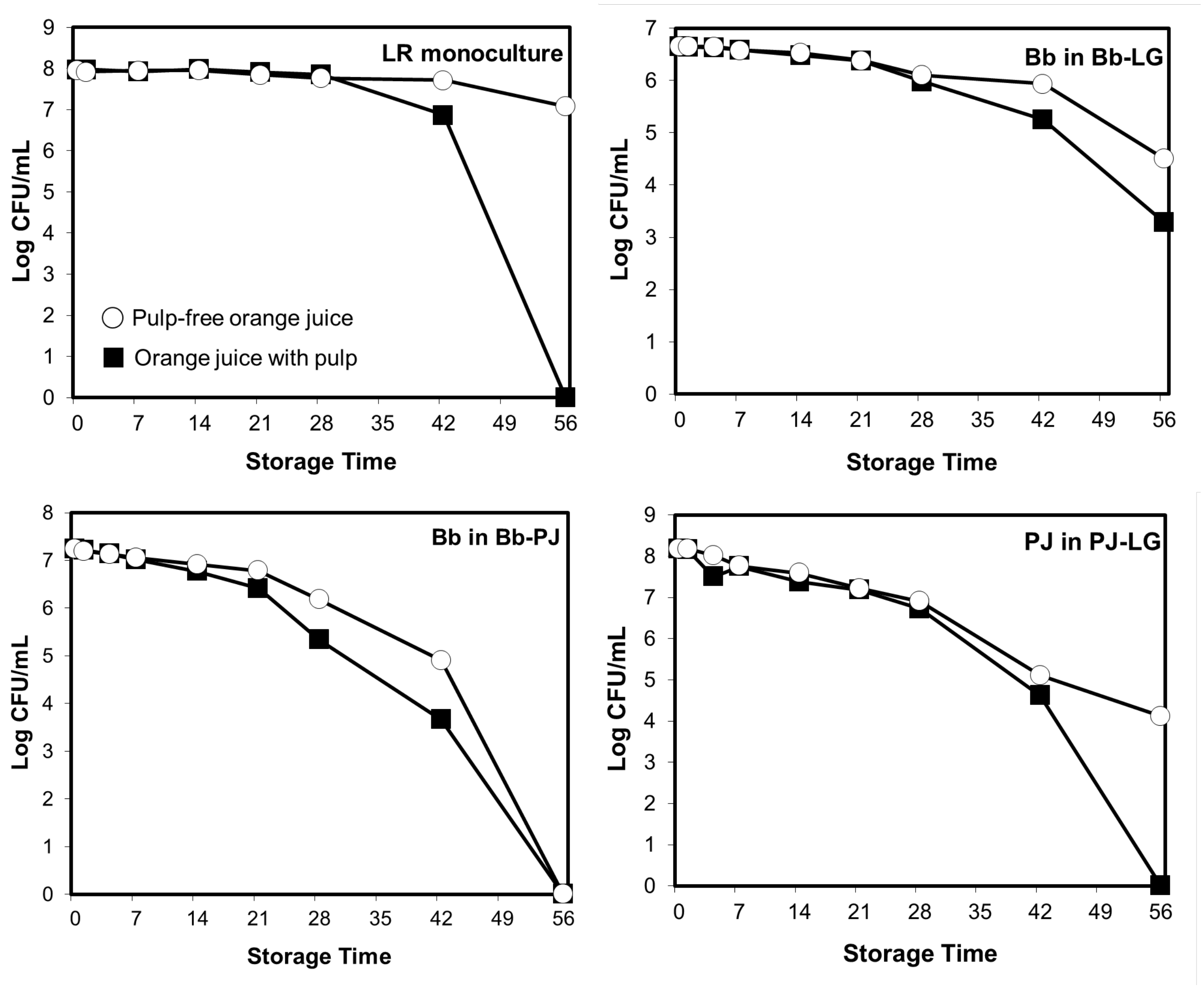

Supplementary Figure S1. Viable cell counts of probiotics included in orange juice with and without pulp over 8 weeks of storage at 4 °C. Only those preparations with significant differences between the individual probiotics included in the orange juice with and without pulp are shown. Due to smaller size of standard error (SE) values (ranging from 0.006 to 0.275 Log CFU mL^{-1}) compared with the size of markers, they are hidden behind the markers.
